# *Helicobacter pylori* in the Oral Cavity: Current Evidence and Potential Survival Strategies

**DOI:** 10.3390/ijms232113646

**Published:** 2022-11-07

**Authors:** Lin Zhang, Xi Chen, Biao Ren, Xuedong Zhou, Lei Cheng

**Affiliations:** 1 State Key Laboratory of Oral Diseases, National Clinical Research Center for Oral Diseases, West China Hospital of Stomatology, Sichuan University, Chengdu 610041, China; 2 Department of Operative Dentistry and Endodontics, West China School of Stomatology, Sichuan University, Chengdu 610041, China

**Keywords:** *Helicobacter pylori*, survival strategy, oral cavity, biofilm, *Candida albicans*

## Abstract

*Helicobacter pylori* (*H. pylori*) is transmitted primarily through the oral–oral route and fecal–oral route. The oral cavity had therefore been hypothesized as an extragastric reservoir of *H. pylori*, owing to the presence of *H. pylori* DNA and particular antigens in distinct niches of the oral cavity. This bacterium in the oral cavity may contribute to the progression of periodontitis and is associated with a variety of oral diseases, gastric eradication failure, and reinfection. However, the conditions in the oral cavity do not appear to be ideal for *H. pylori* survival, and little is known about its biological function in the oral cavity. It is critical to clarify the survival strategies of *H. pylori* to better comprehend the role and function of this bacterium in the oral cavity. In this review, we attempt to analyze the evidence indicating the existence of living oral *H. pylori*, as well as potential survival strategies, including the formation of a favorable microenvironment, the interaction between *H. pylori* and oral microorganisms, and the transition to a non-growing state. Further research on oral *H. pylori* is necessary to develop improved therapies for the prevention and treatment of *H. pylori* infection.

## 1. Introduction

*H. pylori* is a microaerophilic pathogen with a typical spiral or arc form that infects about 50% of the world’s population [1]. Although most *H. pylori*-positive individuals are asymptomatic, long-term infection with *H. pylori* has been associated with the development of gastric malignancies, particularly gastric cancer and gastric mucosa-associated lymphoid tissue lymphoma [2]. Hence, this organism was identified as a class I carcinogen by the World Health Organization in 1994. A combination of at least two diagnostic methods is often required to detect *H. pylori* infection and related diseases. Moreover, the emergence of drug-resistant strains poses considerable challenges to current treatment [3]. Therefore, it is beyond dispute that *H. pylori* is associated with a heavy disease burden worldwide.

It is generally accepted that *H. pylori* transmits between the human population through the oral–oral route, the fecal–oral route, and the gastro–oral route [4,5]. The oral cavity is the first channel for *H. pylori* into the human body, and its function in *H. pylori* infection of the human body generates considerable concerns among researchers. Many scholars have employed molecular techniques, immunological or biochemical approaches, and classic culture techniques to identify oral samples as *H. pylori*-positive [6]. Accordingly, studies have been carried out to detect *H. pylori* infection using saliva samples. Such a non-invasive test might be more acceptable to individuals [3]. In addition, some studies have shown that *H. pylori* in the oral cavity could adversely affect the clinical outcome of eradication therapy [7,8], and oral *H. pylori* is considered a risk factor for the recrudescence of gastric *H. pylori* infection [9]. Consequently, it is hypothesized that the oral cavity might be a potential source for *H. pylori* gastric reinfection [10].

**Figure 1:**
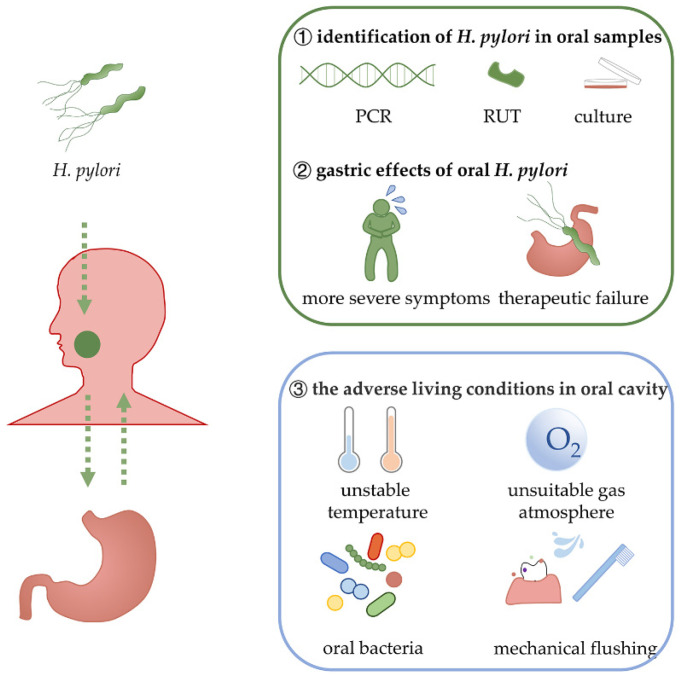
The oral cavity is a potential source for *H. pylori* gastric reinfection. However, the oral cavity seems not to be an ideal habitat for *H. pylori*, owing to the unstable temperature, high O_2_ tension, and varied bacterial composition [13,14]. Therefore, survival strategies of living *H. pylori* in the oral cavity remain to be investigated. The green arrows symbolize the common transmission pathway of *H. pylori*. PCR, polymerase chain reaction; RUT, rapid urease test.

The environment of the oral cavity differs considerably from that of the stomach. It is generally accepted that *H. pylori* is a microaerophilic microorganism that requires high CO_2_ tension to thrive and survive for long periods of time [11,12]. However, owing to the communication with the outside environment, a lower CO_2_ concentration and a higher O_2_ concentration are detected in the oral cavity than in the stomach. The microbial composition, temperature, and mechanical scouring within the oral cavity differ considerably from those of the stomach. For instance, *Streptococcus mitis* (*S. mitis*) and *Streptococcus mutans* (*S. mutans*) may inhibit the growth of *H. pylori* in vitro [13,14]. Furthermore, eating can cause an unstable oral temperature, and the mechanical flushing of saliva might be a barrier to the long-term oral survival of *H. pylori* (Figure 1). Therefore, the inconsistencies between clinical detections and unfavorable conditions represent a challenging question concerning how *H. pylori* adapt to the environment of the oral cavity.

In this review, we analyze various lines of evidence regarding the persistent survival of *H. pylori* in the oral cavity and discuss three potential strategies favoring its survival in order to better comprehend the role of this bacterium in the oral cavity and to inspire the development of adjunctive treatment for improved control of *H. pylori*.

## 2. The Evidence for the Persistent Survival of Oral *H. pylori*

### 2.1. Various Samples from the Oral Cavity Can Be Detected as H. pylori-Positive

*H. pylori* need to reach and continue to survive in the oral cavity, which is considered the first channel of *H. pylori* transmission into the stomach. Many scholars have applied molecular biology and immunology methods to detect oral *H. pylori* in dental plaque [15], saliva [16,17], the tongue coating [18], and dental pulp [19,20,21]. Recently, Sruthi et al. [22] suggested that *H. pylori* can also be detected on deep carious surfaces in the oral cavity of children, with a positivity rate of 70%. In comparison, dental plaque is more likely than saliva to be detected by PCR as *H. pylori*-positive [6], possibly due to the continuous flow of saliva reducing the bacterial load [17]. In a study detecting *H. pylori* in dental pulp, most *H. pylori*-positive specimens from the same teeth remained positive after an interval of one to two weeks, indicating that *H. pylori* colonized the inflamed pulp tissue [20].

Although most studies have reported positive detection of oral *H. pylori* by molecular biology, such results are unsatisfactory because they cannot reflect the viability of *H. pylori* in the oral cavity. Several studies have attempted to culture oral *H. pylori*, indicating that *H. pylori* appears to be an organism that lives in the oral cavity for the long term. In 1989, Krajden et al. [23] successfully isolated and cultured *H. pylori* in dental plaque for the first time; saliva cultures from all 71 patients included in the study failed to show any positive results. Since then, a small number of published studies [6,21] have described the successful isolation and culture of *H. pylori* from dental plaque, saliva, or pulp samples. However, most of these studies confirmed the isolate as *H. pylori* by oxidase test, catalase test, urease tests, or microscopic observations, which cannot rule out the influence of other *H. pylori*-like microorganisms. Therefore, further study with a focus on whole-genome sequencing of the isolated bacterial strain is suggested [6].

### 2.2. The Association between Gastric H. pylori Infection and Oral H. pylori Positivity

Several clinical studies in recent years have reported a close association between gastric *H. pylori* infection and oral *H. pylori* positivity (Table 1). A meta-analysis of 23 studies (1861 patients) found that the rate of coinfection with *H. pylori* in gastric and dental plaque was 49.7% [24]. In theory, the high coexistence rate of *H. pylori* in oral and gastric samples may imply that the oral cavity serves as a reservoir of *H. pylori*. Some scholars further used *H. pylori* virulence marker genes for genotyping and found that the *vacuolating cytotoxin A* (*vacA*) genotype was concordant in 51.1~58% of saliva and biopsy from the same patient, suggested that *H. pylori* strains in that oral cavity and stomach are likely to be homologous [4,25]. However, some scholars found that *H. pylori* was commonly present in the oral cavity with no clear relation to *H. pylori* infection of the stomach [16,26,27], indicating that *H. pylori* in the oral cavity exhibited a degree of independence.

### 2.3. H. pylori in the Oral Cavity Is Associated with Oral Diseases and Gastric Infection

The survival of *H. pylori* in the oral cavity threatens oral health. In recent years, the pathogenic role of oral *H. pylori* has attracted the attention of many researchers. A meta-analysis by Liu et al. [39] showed that oral *H. pylori*, especially in supragingival plaque, is a risk factor for periodontitis. The total proportion of periodontal pathogens in oral *H. pylori*-positive subgingival plaque samples was higher than that in *H. pylori*-negative samples [40], indicating that oral *H. pylori* infection may promote periodontal disease by altering the microecology. Preincubation of *Porphyromonas gingivalis* (*P. gingivalis*) with *H. pylori* affects *P. gingivalis* virulence, including biofilm formation, bacterial internalization into oral keratinocytes, and hemagglutination, indicating that the direct interaction between *P. gingivalis* and *H. pylori* in subgingival plaque may increase the severity or progression of periodontitis [41]. In addition, the expression of periodontitis-related protein Wnt5a and cytokines IL-8, IL-6, and INF-γ was significantly increased after *cagA* + *H. pylori* stimulated the human leukemia mononuclear cell line, suggesting that *H. pylori* can aggravate inflammation progression [40]. In addition to periodontitis, oral *H. pylori* has been reported to be associated with erosive oral lichen planus (OLP) [42,43] and oral squamous cell carcinoma (OSCC) [44]. However, wing to the heterogeneity in interstudy design, conflicting findings have been reported [44,45,46], and the pathogenic role of oral *H. pylori* in these diseases remains unclear. In the next step, detection methods with high sensitivity and specificity should be used to clarify the role and specific mechanism of oral *H. pylori* in the development of oral diseases.

Oral *H. pylori* increases the severity of gastric infections and the difficulty of eradication. On the one hand, oral *H. pylori* was linked to an increased incidence of grade II gastroesophageal reflux, esophageal sphincter relaxation, and duodenitis in a case–control study including 567 patients [7]. On the other hand, the gastric *H. pylori* eradication success rate was significantly lower in oral *H. pylori*-positive patients than in oral *H. pylori*-negative patients (52.2% vs. 91.6%, respectively, *p =* 0.0028) four weeks after eradication therapy [8]. The association between oral *H. pylori* and gastric infection suggests that the oral cavity may be the source of gastric reinfection.

### 2.4. Effects of Oral Hygiene Management on H. pylori Infection

Given the disparity in eradication success rates between gastric *H. pylori* and oral *H. pylori* eradication therapy (85.8% vs. 5.7%, respectively, OR 55.59, *p* < 0.00001) [47], eradicating oral *H. pylori* with systemic therapy remains difficult in ordinary clinical applications [48]. In such cases, adjuvant topical treatment to eradicate oral *H. pylori* seems necessary (Table 2). For instance, eradication therapy combined with periodontal therapy can increase the eradication rate of gastric *H. pylori* [49,50]. Consistently, the OR for the unsuccessful gastric eradication increased 64-fold if periodontal treatment failed to eliminate oral *H. pylori* [51]. Oral hygiene management can reduce *H. pylori* in the oral cavity and help to control its migration from to the stomach and can be used as an adjuvant treatment option for gastric *H. pylori* eradication therapy. Attention should be paid to the development of oral healthcare products with anti-*H. pylori* effect to contribute to oral defense against *H. pylori*.

The evidence presented thus far supports the idea that *H. pylori* is likely to survive in the oral cavity and be involved in gastric infection. However, currently, we can only reduce the negative effects of oral *H. pylori* by some oral hygiene treatments. Therefore, further investigation of the details of oral *H. pylori* survival is required to achieve the effective eradication of *H. pylori* infection. 

## 3. Pangenome and Virulence Factors

The prevalence of *H. pylori* infection is not always correlated with the incidence of gastric diseases. Only a small percentage of those infected progress to peptic ulcers or even gastric cancer. Some countries in Africa and Asia have lower rates of gastric cancer and higher rates of *H. pylori* infection, whereas others exhibit the opposite trend. Such a phenomenon of clinical diversity is determined by the genetic variability of the infecting *H. pylori* strains; the genetic background and immunity of different ethnic groups; gastric and intestinal microbiota; and environmental factors, such as geographic location and dietary habits [55,56,57,58]. For example, *H. pylori* infection status can change oral microorganism composition and alter the interactions between microorganisms, whereas the oral host–microbial interactome could provide signals to impact health and disease [59]. The oral microbiota of the host may cause carcinogenesis via various potential mechanisms, including the induction of chronic inflammation, the inhibition of the host’s immune system, antiapoptotic activity, and the production of carcinogenic substances that may fuel the progression of cancer [60].

Geographically stratified *H. pylori* subpopulations have emerged as a result of the mode of intrafamilial transmission and long-term coevolution with human hosts [61]. The whole-genome sequencing of *H. pylori* indicated a high rate of gene recombination and unusual genetic flexibility; these traits enable the bacteria adapt to the dynamic environment [62,63]. Therefore, systematic analysis of the whole-gene repertoire, termed the pan-genome, is important for understanding bacterial intraspecies diversity, population genetics, and evolution. Sequence-alignment-based multilocus sequence typing (MLST) of *H. pylori* grouped *H. pylori* isolates into seven types connected to geographic information [64], and the conserved regions and genes among *H. pylori* genomes were potentially associated with *H. pylori* pathogenicity and adaptation, such as *cag* pathogenicity island (*cag*PAI) [62,63]. A pangenome Fst analysis showed that variation in virulence genes was more common in the Americas than other regions [65]. A total of 22 of the 35 genes with the highest Fst values encode recognized virulence factors and membrane proteins, suggesting that virulence plays a strong role in *H. pylori* adaption to specific human populations [65].

*H. pylori* virulence factors can be categorized to be related with three main pathogenic steps: bacterial colonization, immune evasion, and disease induction [66]. The helical shape and flagella enable *H. pylori* to penetrate the mucous layer and subsequently adhere to cellular surface receptors via adhesins [67]. Urease hydrolyses urea, thus neutralizing acidic pH and forming a neutral layer favorable to *H. pylori* survival [67]. The blood group antigen-binding adhesion (BabA) and sialic acid-binding adherence (SabA) are two of the most extensively studied *H. pylori* outer-membrane proteins, which function as adhesins that mediate *H. pylori* binding to gastric epithelial cells [68]. The surface features and various virulence proteins have been identified to contribute to immune evasion and disease induction. 

CagA and VacA are the main *H. pylori* virulence factors involved in immune evasion and disease induction and are likely to cause disease in the oral cavity [69]. *cag*PAI is a 35–40 kb DNA segment located on the *H. pylori* chromosome and carries more than 30 genes. The variability of *H. pylori* is reflected in the frequency of possession of the *cag*PAI, as the carriage of *cag*PAI varies from almost universal presence in hpEastAsia and hpAfrica1 through intermediate presence (hpEurope) to complete absence (hpAfrica2) [70]. *cag*PAI encodes an antigenic effector protein (CagA). The odds ratio for dysplasia was reported to be higher in cagA-positive individuals compared with cagA-negative individuals (15.4 vs. 0.90, respectively), suggesting that *cagA* is associated with increased gastric cancer risk [71]. The Western-type CagA and East Asian-type CagA were further described based on the repeat sequence Glu-Pro-Ile-Tyr-Ala (EPIYA) motifs at the N-terminus of CagA and their binding activity to Src homology 2 (SH2)-containing tyrosine phosphatase SHP-2, whereas East Asian-type CagA was reported to confer stronger SHP-2 binding and morphologically transforming activities compared to Western CagA, making East Asian-type CagA more virulent [67,72]. In addition, genes encoding proteins of type IV secretion systems (T4SS) are located in cagPAI. CagA can be delivered into the cytoplasm via the T4SS and can subsequently interact with at least 10 host cell components in both a phosphorylation-dependent and phosphorylation-independent manner, altering the cellular signal transduction system [73]. *cag*+ *H. pylori* strains also showed diversity in terms of levels of CagA production, whereas strains producing higher levels of CagA were associated with increased risk of premalignant lesions [74,75].

VacA is another extensively studied virulence factor that can be produced by all *H. pylori* strains. VacA can induce the formation of large cytoplasmic vacuoles in host cells and is involved in immune evasion of *H. pylori*. However, there are differences between strains at the level of VacA production or secretion and in the amino acid sequence among VacA proteins [76]. Several vacA subtypes can be divided according to combinations of the signal sequence (s1a, s1b, s1c, and s2), mid-region (m1, m1T, and m2), and the intermediate region (i1, i2, and i3) [67]. As a result, infection with strains carrying combinations of these hypervirulence genes (e.g., *vacAs1m1cagA* +) is associated with a higher risk of invasive disease than infection with strains carrying combinations of less virulent alleles (e.g., *vacAs2m2cagA*−) [57]. 

The genetic diversity of *H. pylori* may be associated with their adaptation to the host and disease progression. Attention should be paid to the virulence characteristics and genetic background of oral *H. pylori*. Further pangenomic analysis of the genes related to *H. pylori* colonization can facilitate an improved understanding of the survival strategies of *H. pylori* in the oral cavity.

## 4. Interactions between *H. pylori* and Microenvironments in the Oral Cavity

The oral cavity might be the prime habitat for *H. pylori* colonization and gastric re-infection; however, how *H. pylori* adapt to the environment of the oral cavity is still unclear. The mutual interaction of *H. pylori* with the local oral environment can be considered on two levels. The first focuses on biofilm formation; the biofilm matrix plays a synergistic role in protecting *H. pylori* against unfavorable factors. The second concerns the physical contact with host cells, whereby the adhesion and invading capability of *H. pylori* may favor its oral survival. 

### 4.1. Oral Microenvironment and H. pylori

As mentioned above, *H. pylori* has been detected in plaque samples collected from carious cavities and periodontal pockets, suggesting that it can survive in the microenvironments of these niches, where biofilm provides various benefits for bacterial reproduction, metabolism, and defense and is the favored method of long-term survival for many colonizers. Therefore, *H. pylori* is often found in dental plaque, where the detection rate is often higher than that in saliva. The layered morphology of biofilm suggests that there can be gradients in nutrients, gas concentration, and pH value. When observed at the micron scale, supragingival plaque has a complex microbial consortium called “hedgehog” composed primarily of *Corynebacterium*, a multitaxon filament-rich annulus, and a periphery of *Streptococcus*-containing corncob structures [77,78]. In corncob structures, *Streptococcus* cells consume carbohydrates and O_2_ to generate CO_2_, thus generating a CO_2_-rich environment for survival of microaerophilic and anaerobic microbes, which might be favorable to *H. pylori* survival. Typically, cariogenic microorganisms in dental plaque produce acids upon exposure to carbohydrates, resulting in a decrease in pH value, which challenges the survival of *H. pylori*. Oral *H. pylori* also needs to adapt to the changing pH value of cariogenic dental plaque. In this case, urea-metabolizing capacity is important for the survival of alkali-producing symbionts in dental plaque [79], and the same may also hold true for *H. pylori*, which can use urease to convert urea into ammonia and CO_2_ with a certain buffering capacity, leading to medium alkalization around itself [57]. The formation of biofilms in these niches enables the long-term adhesion of *H. pylori* without being affected by saliva flushing or food chewing. Moreover, bacteria surviving in biofilms are usually resistant to host defense systems and antimicrobial medicines, protecting them from the adverse effects of drugs during systemic treatment [80,81], which may be among the reasons why it is so difficult to eliminate oral *H. pylori*. 

### 4.2. Oral Host Cells and H. pylori

Interaction with the host is also an important strategy for *H. pylori* survival in dental pulp. *H. pylori* surviving in biofilm may reach pulp through caries cavities and survive in dental pulp, as pulp *H. pylori*-positive teeth often have deep cavities, whereas those with milder caries are rarely detected with pulp *H. pylori* positivity [21,82]. For instance, *H. pylori* has the ability to adhere to host cells in dental pulp. *H. pylori* ATCC 51932, *H. pylori* 26695, and *H. pylori* J99 all displayed adhesion capabilities in human dental pulp fibroblast cells (HDPFs), whereas the other two *cagA*-positive strains showed higher adhesion rates than *cagA*-negative *H. pylori* ATCC 51932 [20]. In studies involving gastric epithelial cells, CagA (cytotoxin associated antigen) was reported to disrupt host cell polarity, enabling adherent *H. pylori* to replicate and grow on the cell surface [83,84]. 

*Helicobacter* outer-membrane protein Q (HopQ) was recently found to bind to the receptor carcinoembryonic antigen-related cell adhesion molecule family (CEACAMs) exposed on the host cell surface [85,86]. Consequently, this interaction allows bacteria to adhere to host cells and is required for the injection of CagA into host cells via the type IV secretion system (T4SS) [85,86]. However, unlike gastric epithelial cells, oral epithelial cell-derived cell lines HN, CAL-27, and BHY were reported to be resistant to CagA injection due to the lack of CEACAM expression, suggesting that healthy oral epithelium cells may lack an *H. pylori* docking site [87]. However, the *ceacam1* gene is highly upregulated during palate development [88]. Moreover, CEACAM expression in the oral cavity is upregulated in patients with oral cancer, periodontitis, and oral lichen planus, as well as in smokers [89,90,91,92]. Therefore, in some pathological conditions, the increased expression of oral CEACAMs may favor *H. pylori* adhesion to oral host cells and even create favorable conditions for CagA injection into oral cells, inducing inflammatory factors. Unfortunately, no in vivo evidence exists to support these ideas. 

In addition, CEACAM1 functions as an inhibitory receptor on various immune cells, including T and NK cells [93]. *H. pylori* HopQ can inhibit interferon-gamma (IFN-γ) secretion of CD4 cells and suppress T or NK cell cytotoxicity by interacting with CEACAM1, and the inhibition of immune cells may help protect developing gastric tumors from immune cell attack [94]. For *H. pylori* survival, this inhibitory effect may also benefit the peaceful coexistence of the *H. pylori* immune system, which is consistent with the fact that the majority of *H. pylori*-positive individuals are asymptomatic. The interaction of *H. pylori* with human immunity is discussed further in Section 4.3.

*H. pylori* is able to invade gastric epithelial cells and complete the entire biological cycle, including proliferation and apoptosis, within the cells [95,96,97]; a similar phenomenon has been observed in the oral cavity. Coccoid forms of *H. pylori* SS1 were localized and surrounded by vacuoles in the cytoplasm of human periodontal ligament fibroblasts (hPDLFs), and the invasion of *H. pylori* SS1 can adversely affect basic cellular functions of hPDLFs, resulting in G2 phase arrest and inhibition of cell proliferation [98]. Interestingly, *H. pylori* can also invade immune cells and survive within them by affecting autophagy [99,100]. This intracellular location could facilitate *H. pylori* evasion of host immune surveillance and antibiotic pressure, allowing *H. pylori* to intracellularly persist, proliferate, and spread to adjacent tissues. At present, it is still unclear whether invading *H. pylori* can be released from the cells, and more studies are necessary to establish the effects on cell functions.

### 4.3. Human Host Immunity and H. pylori

The host immune system can produce antimicrobial peptides, activate the cellular autophagy pathway, and enhance oxidative stress against *H. pylori* infection [73]. The survival tactics of *H. pylori* in stomach mucosa have been well-studied; one of the strategies of *H. pylori* is to modulate surface features that interfere with host immune system recognition [101]. For example, the negatively charged group on *H. pylori* lipopolysaccharide (LPS) is replaced, which reduces the surface negative charge and thus resists cationic antimicrobial peptide action [102]. LPS from *H. pylori* is less able to activate the Toll-like receptor (TLR), and a recent study revealed that *H. pylori* specifically binds human annexins via lipid A and strongly inhibits LPS-mediated TLR4 signal transduction to avoid its recognition by the innate immune system [103].

As mentioned previously, HopQ-CEACAM interaction suppresses immune cell function. In addition, *H. pylori* controls the production and secretion of chemokines in immune cells via HopQ-CEACAM interaction and survives within neutrophils in a HopQ-dependent manner [104]. The classically activated macrophages (M1 macrophages) highly express proinflammatory cytokines IL-1β, transforming growth factor-β (TNF-β), and nitric oxide synthase (iNOS), leading to the reduction in bacterial load and enhanced pathology [105]. To achieve long-term survival in the host, *H. pylori* has been found to inhibit macrophage phagocytosis and suppress T-cell activation by hindering expression of human leukocyte antigen-II (HLA-II) and IFN-γ production from macrophages [106]. The expression of programmed cell death ligand 1 (PD-L1) has been suggested as an immune modulatory mechanism for persistent infection of *H pylori*, and dendritic cells expressing higher levels of PD-L1 have been found to impede *H. pylori*-induced inflammation but allow persistent *H. pylori* colonization in mice [107].

*H. pylori* virulence factors are often considered to contribute to the progression of gastric pathology. However, from the bacterial perspective, the virulence factors may be mechanisms supporting escape from host immune clearance and maintenance of chronic infection [108]. For example, VacA is able to suppress phagocytosis, induce tolerogenic dendritic cells, and block effector T-cell response, thus inhibiting the function of various immune cells [66]. In addition, *H. pylori* can affect cell autophagy via VacA to benefit its intracellular survival [73]. The interaction between *H. pylori* and host immunity may influence the success of *H. pylori* colonization. Because *H. pylori* is often detected in infected dental pulp and periodontal pockets, the interaction between *H. pylori* and local immunity warrants further research.

## 5. The Synergistic Interactions of Oral *H. pylori* with Oral Microorganisms

Clinically, people with caries or periodontitis are more likely to be infected with oral *H. pylori* [26,109,110]. Survival of *H. pylori* in the oral niches may benefit from interactions with oral microorganisms in these lesions, such as *S. mutans* [111,112], *Candida albicans* (*C. albicans*) [113], *P. gingivalis* [114], and *Fusobacterium nucleatum* (*F. nucleatum*) [115] (Table 3).

*S. mutans* is the most common cariogenic microorganism; there may be a synergistic relationship between *S. mutans* and *H. pylori. H. pylori* can grow throughout the biofilm formed by *S. mutans* in vitro, and the location of *H. pylori* in biofilms was reported to be dependent upon the presence or absence of *S. mutans* [111]. *Streptococcus* in biofilm contributes to the generation of a CO_2_-rich environment, which might be related to *H. pylori* benefiting from *S. mutans* in biofilm coculture. Moreover, the production of mutant proteins and the acid of *S. mutans* could be induced by *H. pylori* supernatant, with *S. mutans* showing a competitive advantage over *S. sanguinis*, indicating that *H. pylori* could create favorable conditions for *S. mutans* [112]. Based on these existing studies reporting positive feedback between *S. mutans* and *H. pylori*, *S. mutans* could provide a colonization environment for oral *H. pylori*, whereas oral *H. pylori* can create a competitive advantage for *S. mutans*.

*P. gingivalis* and *F. nucleatum* are the main anaerobic pathogens of periodontal disease. *H. pylori* is known to have the ability to coaggregate with *P. gingivalis*, which can facilitate the long-term persistence of *H. pylori* in periodontal pockets [14]. *H. pylori* may promote the severity of periodontitis. Preincubation of *P. gingivalis* with *H. pylori* enhanced *P. gingivalis* virulence, including biofilm formation, bacterial internalization into oral keratinocytes, and hemagglutination [41]. In addition, the role of *F. nucleatum* in the survival of *H. pylori* is worthy of further investigation, as *H. pylori*-negative chronic gastritis patients were reported to have lower levels of *F. nucleatum* in saliva than healthy subjects [124]. Generally, *F. nucleatum* plays a critical role in the formation and maturation of dental plaque biofilms, owing to its long and narrow rod-like structure and the expression of a variety of adhesins [125,126]. *H. pylori* adheres to *F. nucleatum* and thus colonizes dental plaque through coaggregation [14,115]. Therefore, *F. nucleatum* may act as a bridge and make an important contribution to the long-term survival of *H. pylori* in oral biofilms. 

However, oral organisms such as *S. mutans* and *Prevotella intermedia* (*P. intermedia*) can also inhibit the growth of *H. pylori* during coculture in vitro [14,123]. The intricate and dynamic interactions between *H. pylori* and oral microorganisms might make in vitro cultivation of *H. pylori* difficult. 

Fungus is another important component of the oral microbiome. *C. albicans* is one of the most prevalent fungi in humans involved in oral infectious diseases. Clinical studies have reported the coexistence of *C. albicans* and *H. pylori* in the vagina and stomach [127,128]. Complex cross-kingdom interactions occur between the two organisms [113,116]. Sánchez-Alonzo et al. [116] observed an accumulation of coccoid and bacillary bacteria on yeast pseudohyphae cocultured with *H. pylori*. Furthermore, several surface interaction mechanisms, including hydrophobic interactions between non-polar peptide chains and lipid structures, hydrogen bonds, and thiol-mediated surface interactions, occur between *H. pylori* and *C. albicans*, eventually contributing to the formation of polymicrobial biofilms [117]. These synergistic interactions may be related to *C. albicans* in mixed-species biofilm consuming oxygen to support the milieu changes from aerobic to anaerobic, favoring the growth of anaerobes [129]. 

*H. pylori* can invade the *C. albicans* yeast cells and was strained green by live/dead BacLight staining, indicating that *H. pylori* can survive within *C. albicans* [118,119]. Consistent with this phenomenon, fast-moving bacteriolar-like bodies (BLBs) were observed within the vacuoles of the *C. albicans* yeast cells and were subsequently identified as *H. pylori* using PCR and fluorescence in situ hybridization (FISH) techniques [122,130]. Some non-adaptive conditions, such as nutrient deprivation [116], acidic pH [121], amoxicillin [120], and other stress factors, can further induce *H. pylori* to invade yeast cells. As a consequence, the invading *H. pylori* can survive unfavorable factors, such as high temperature, desiccation, and antibiotic exposure while expressing proteins [131] and showing an active motility state in vacuoles [120]. *H. pylori*-carrying *C. albicans* has been found in food [132] and in many other ecological niches, such as the human oral cavity [133] and vagina [134]. Therefore, yeast cells may provide *H. pylori* with an intracellular niche that protects *H. pylori* from unfavorable conditions.

Interestingly, *Candida* not only harbors intracellular *H. pylori* but also contributes to the transmission of *H. pylori*. Invading *H. pylori* can propagate vertically to the vacuoles of daughter cells of yeasts in consecutive subcultures of yeasts [118]. Moreover, vesicle-encased or free *H. pylori* can be released by *C. albicans*, and the released *H. pylori* may invade new *C. albicans* yeast cells [122]. In addition to transmission between yeast cells, the *H. pylori* can spread within yeast cells to various human bodies and niches. For instance, normally born babies had a higher frequency of *H. pylori*-invaded *C. albicans* in the oral cavity than babied born by cesarean birth [128,134], indicating that *H. pylori*-carrying *C. albicans* in the vagina may support *H. pylori* transmission to newborns. As a result, reducing the yeast content of the oral cavity might be beneficial in terms of controlling the infection and transmission of oral *H. pylori* [122]. 

In conclusion, *H. pylori* interacts with various oral microorganisms to survive in the oral cavity for a long time and can even invade *C. albicans* yeast cells to protect itself from harsh conditions. The antagonistic relationship and the invasion of *H. pylori* into yeast cells may lead to difficulties associated with isolating and culturing *H. pylori* in vitro. 

## 6. Non-Growing State of *H. pylori*: Viable but Non-Culturable State and Dormant State

Despite the existence of some synergistic factors, the oral environment is complex and hostile. Non-sporulating bacteria can transmit to the viable but non-culturable (VBNC) state when exposed to harmful stimuli. In the VBNC state, bacterial cells are reduced in size and metabolic activity and become unculturable in vitro [135]. However, the transformation to the VBNC state is reversible, and several VBNC bacterial cells were able to recover to a culturable state under specific conditions [135]. 

When cultured in the laboratory, *H. pylori* transform into a spherical shape and lose of culturability under various adverse conditions, such as anaerobic culture [136], nutrient deprivation [137,138], and long-term liquid culture [139,140]. However, there is evidence that these unculturable *H. pylori* are capable of active transcription and translation processes [136,138,141,142,143,144], possibly cells in the VBNC state. VBNC *H. pylori* cells have been found to be distributed in freshwater and seawater [139,143,145,146], but no resuscitation technology has been found for *H. pylori* in vitro [139]. 

*H. pylori* in the VBNC state also has certain pathogenic abilities. *H. pylori* and physiological changes were detected in mouse stomachs after inoculation of VBNC *H. pylori* suspension, suggesting that VBNC *H. pylori* can colonize the gastric wall of mice and induce mucosal tissue damage, although less virulently than helical *H. pylori* [147]. However, there appear to be differences in the infectivity of VBNC *H. pylori* of different strains. For instance, *H. pylori* SS1 in the VBNC state in drinking water was unable to infect mice [148]. In another study, *H. pylori* strain 553/93 in the VBNC state produced more severe inflammation than the other two tested VBNC strains [149]. 

Another common survival strategy under adverse conditions is dormancy. In a stressful environment (e.g., pH and temperature changes, nutrient deficiencies, and antimicrobial drugs), microorganisms can escape threats by reversibly transitioning from an active state to an inactive (dormant) state [150,151]. In the dormant state, bacteria exhibit little or low metabolic activity [152], remain unreplicated for long periods of time, and have increased resistance to extreme stress [150]. Dormant and VBNC states are similar but differ in terms of performance, which has led to some controversy in distinguishing the two. Some scholars believe that in both the VBNC state and the dormant state, bacteria can survive under adverse conditions but cannot be cultured. Therefore, the two terms can be used to describe the same physiological state; alternatively, the VBNC state is a type of dormant state [150]. Other scholars believe that bacteria still exhibits a certain metabolic activity in the VBNC state, which should be distinguished from the dormant state that does not exhibit obvious metabolic activity [153]. 

Some bacteria in the oral cavity may have an antagonistic relationship with *H. pylori* [154], and parts of biofilm have suboptimal growth conditions (e.g., reduced nutrient concentrations or acidity, hypoxia) [79,155], thereby promoting the transition of *H. pylori* to a VBNC or dormant state. VBNC or dormant *H. pylori* are highly resistant to adverse environments, which may contribute to the survival of oral *H. pylori*. Few isolated cultures of oral *H. pylori* can be associated with these non-growing states. Although *H. pylori* coccoid, similar to dormant or VBNC states, have been found in oral samples [21,98], the exact status of this coccoid form of *H. pylori* and its role in survival are still unclear. At present, little is known about the physiological changes and survival status of oral *H. pylori*. 

## 7. Conclusions

**Figure 2:**
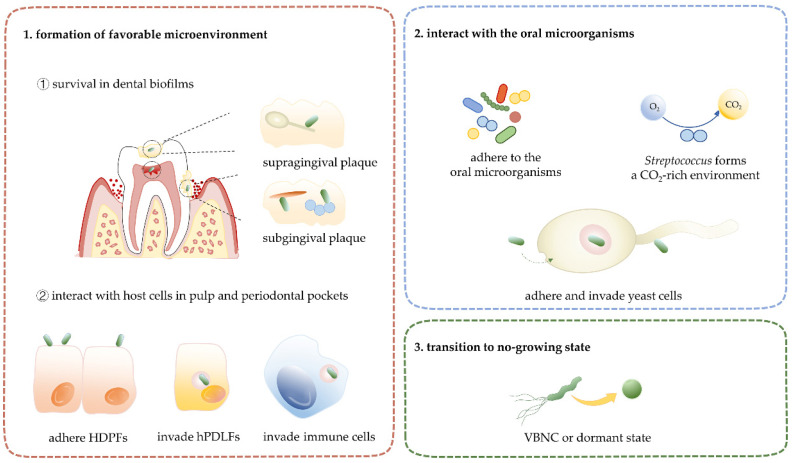
Potential survival strategies of *H. pylori* in the oral cavity. *H. pylori* can hide within dental plaque biofilm in caries cavities and periodontal pockets. Moreover, this organism has the ability to adhere to and invade host oral cells in these niches. Synergistic interaction with oral microorganisms and transition to a VBNC or dormant state may also help *H. pylori* adapt to adverse conditions in the oral cavity.

Numerous studies have shown the existence of *H. pylori* in a variety of oral niches, including dental plaque, infected pulp, and periodontal pockets, implying that *H. pylori* may be able to survive in the oral environment through certain survival strategies. The formation of biofilms in these niches enables the long-term adhesion of *H. pylori* without being affected by saliva washing or food chewing. In addition, *H. pylori* can adhere to and invade host cells in the oral cavity. Furthermore, *H. pylori* can coaggregate with a variety of oral bacteria and yeast cells, and the invading *H. pylori* is able to escape from some extracellular pressure. Additionally, transitioning to a non-growing state may be another important strategy for *H. pylori* to adapt to unfavorable oral cavity conditions, which, together with invading cells and growth inhibition by other microorganisms, explains why oral *H. pylori* is difficult to culture (Figure 2).

## Figures and Tables

**Table 1:** Summary of data regarding *H. pylori* coinfection in gastric biopsy and oral samples.

Oral Sample	Sample Size	*H. pylori* Detection Method in Oral Samples	Coinfection Rate	Ref.
Saliva	689 *H. pylori*-associated gastritis patients	PCR-16S rRNA and *ureA* gene	79.7%	[28]
Saliva	162 patients with gastric disease	PCR	24%; 51.1% agreement in *vacA* genotype in saliva and biopsy from the same patients	[25]
Saliva	300 patients with gastric disease	PCR-*ureC*, *cagA*, and *vacA* gene	10.72%; high homology (58%) in *vacA* genotype in saliva and gastric samples from the same patients	[4]
Dental plaque	235 patients with chronic gastritis	PCR-16S rRNA	56.52%	[29]
Dental plaque	164 dyspeptic patients	Enzyme Immunoassay	82.1%	[30]
Dental plaque	65 patients with gastric *H. pylori* infection among 134 dyspeptic patients	RUT	89.2% among gastric *H. pylori*-positive patients	[31]
Subgingival plaque	101 dyspeptic patients	RUT	66%	[32]
Subgingival plaque	443 dyspeptic patients	Nested PCR- 860bp fragment	71.86%	[33]
Subgingival plaque	67 dyspeptic patients	PCR-*ureA* gene	25.4%	[34]
Dental plaque and saliva	70 children with dyspepsia	PCR-16S rRNA and 23S rRNA	Dental plaque (77.6%); saliva (75.9%)	[26]
Dental plaque and saliva	61 patients with dyspepsia	PCR-*ureA* gene	50.8%; 38.7% genotype concordance between oral and gastric samples from the same patients	[35]
Dental plaque and saliva	62 patients with dyspepsia	PCR-16S-rRNA	All oral samples (68%)	[36]
Dental plaque and saliva	30 patients with gastric disease	PCR-*cagA* gene	60%; 98% agreement between gastric DNA *H. pylori* sequence and their corresponding saliva or dental plaque DNA	[37]
Supragingival plaque, subgingival plaque, and saliva	56 gastric *H. pylori*-positive patients with periodontitis	PCR-16S rDNA	All oral samples (41.1%); supragingival plaque (26.8%); subgingival plaque (30.4%); saliva (21.4%);	[38]

*ureA*, α-subunit of the urease gene; *cagA*, cytotoxin-associated gene A; *vacA*, vacuolating cytotoxin A.

**Table 2:** Oral hygiene management strategies against *H. pylori* infection.

Oral Hygiene Management	Eradication Therapy	Effects on Gastric *H. pylori* Infection	Ref.
Scaling and/or combined with root planing and oral hygiene instructions on brushing with the modified Bass technique	10-day course of triple therapy consisted of a PPI combined with amoxicillin (2 × 1 g daily) and clarithromycin (2 × 500 mg daily)	The eradication rate in the combined therapy group was higher than that in the triple therapy only group (64.71% vs. 51.06%, respectively, *p* = 0.17).	[51]
Scaling and root planing; oral hygiene instruction	1-week triple therapy (esomeprazole 20 mg twice per day, clarithromycin 500 mg twice per day, or metronidazole 400 mg three times per day (if clarithromycin-resistant), as well as amoxicillin 1000 mg twice per day)	The recurrence rate of gastric *H. pylori* in the combined therapy group was lower than that in the triple therapy only group (2.04% vs. 15.27%, respectively; OR 0.69; 95% CI 0.52 to 0.99; *p* = 0.001).	[28]
Mouth rinse (0.02% tinidazole and 0.12% chlorhexidine) with 20 mL held in the mouth for 5 min for 10 d; ultrasonic periodontal scaling twice a month	Triple therapy consisted of amoxicillin (1.0 g) and esomeprazole (20 mg) twice a day and levofloxacin (0.5 g) once a day for 10 d	The eradication rate in the combined therapy group was higher than that in the triple therapy only group (94.7% vs. 78.4%, respectively, *p* = 0.012).	[52]
Basic periodontal therapy during triple therapy	7-day course of triple therapy consisted of amoxicillin 2 g/day (g/d), clarithromycin 1 g/d, and pantoprazole 80 mg/d	The eradication rate in the combined therapy group was higher than that in the triple therapy only group (77.3% vs. 47.6%, respectively, *p* = 0.044).	[53]
Oral hygiene education, dental cleaning, and scaling	14-day PPI or triple therapy	The eradication rate in the combined therapy group was higher than that in triple therapy only group (62.8% vs. 32.4%, respectively, *p* < 0. 05).	[54]

**Table 3:** Interaction between *H. pylori* and oral microorganisms.

Interaction Type	Oral Microorganisms	Interaction between *H. pylori* and Oral Microorganisms	Ref.
Mutualistic relationship	*S. mutans*	*H. pylori* can penetrate the biofilm formed by *S. mutans*.	[111]
		*S. mutans* contributes to the formation of “hedgehog” in the dental plaque, which could generate a CO_2_-rich environment.	[77]
	*F. nucleatum*	*H. pylori* can adhere to *F. nucleatum* and might help to colonize the dental plaque.	[15,115]
	*P. gingivalis*	*H. pylori* has the ability to coaggregate with *P. gingivalis*.	[14]
		*P. gingivalis* with a specific *filamentous appendage* (*fimA*) genotype may be involved in the colonization by *H. pylori*.	[114]
	*C. albicans*	*H. pylori* can adhere to yeast pseudohyphae.	[116]
		*H. pylori* can anchor on *C. albicans* and form polymicrobial biofilms.	[117]
		*H. pylori* can invade yeast cells.	[118,119]
		Nutrient deprivation, acidic pH, and amoxicillin may stimulate the entry of *H. pylori* into *Candida*.	[116,120,121]
		*H. pylori* entering yeast cells can propagate vertically to the vacuoles of progeny yeast cells.	[118]
		*C. albicans* releases *H. pylori* as a vesicle-encased or free bacterium, which may facilitate *H. pylori* invasion of new yeast cells.	[122]
Antagonistic relationship	*S. mitis*	The diffusible factors released by *S. mitis* can inhibit the growth and induce the coccoid conversion of *H. pylori* during coculture in vitro.	[13]
	*S. mutans*, etc.	Bacteriocin-like inhibitory proteins against *H. pylori* could be produced by oral bacteria.	[14]
	*S. mutans* and *Prevotella intermedia*	Culture supernatants of these bacteria showed growth inhibitory activity against *H. pylori* and caused the formation of the coccoid form of *H. pylori* in vitro.	[123]

## Data Availability

Not applicable.
